# 504 Single Center Outcome Comparisons of Skin Disorders Versus Burns: Time to Reconsider the Automatic Transfer?

**DOI:** 10.1093/jbcr/irad045.101

**Published:** 2023-05-15

**Authors:** Jamie Hollowell, Jason Nam, Robert Matthew, Booker King, Rabia Nizamani, Felicia Williams, Joyce Pak, Chris Agala

**Affiliations:** UNC Jaycee Burn Center, Chapel Hill, North Carolina; Duke University Hospital, Durham, North Carolina; UNC Chapel Hill, Chapel Hill, North Carolina; UNC Jaycee Burn Center, Chapel Hill, North Carolina; UNC Jaycee Burn Center, Chapel Hill, North Carolina; University of North Carolina at Chapel Hill, Chapel Hill, North Carolina; University of North Carolina Chapel Hill, Chapel Hill, North Carolina; UNC Chapel Hill, Dept of Surgery, Chapel Hill, North Carolina; UNC Jaycee Burn Center, Chapel Hill, North Carolina; Duke University Hospital, Durham, North Carolina; UNC Chapel Hill, Chapel Hill, North Carolina; UNC Jaycee Burn Center, Chapel Hill, North Carolina; UNC Jaycee Burn Center, Chapel Hill, North Carolina; University of North Carolina at Chapel Hill, Chapel Hill, North Carolina; University of North Carolina Chapel Hill, Chapel Hill, North Carolina; UNC Chapel Hill, Dept of Surgery, Chapel Hill, North Carolina; UNC Jaycee Burn Center, Chapel Hill, North Carolina; Duke University Hospital, Durham, North Carolina; UNC Chapel Hill, Chapel Hill, North Carolina; UNC Jaycee Burn Center, Chapel Hill, North Carolina; UNC Jaycee Burn Center, Chapel Hill, North Carolina; University of North Carolina at Chapel Hill, Chapel Hill, North Carolina; University of North Carolina Chapel Hill, Chapel Hill, North Carolina; UNC Chapel Hill, Dept of Surgery, Chapel Hill, North Carolina; UNC Jaycee Burn Center, Chapel Hill, North Carolina; Duke University Hospital, Durham, North Carolina; UNC Chapel Hill, Chapel Hill, North Carolina; UNC Jaycee Burn Center, Chapel Hill, North Carolina; UNC Jaycee Burn Center, Chapel Hill, North Carolina; University of North Carolina at Chapel Hill, Chapel Hill, North Carolina; University of North Carolina Chapel Hill, Chapel Hill, North Carolina; UNC Chapel Hill, Dept of Surgery, Chapel Hill, North Carolina; UNC Jaycee Burn Center, Chapel Hill, North Carolina; Duke University Hospital, Durham, North Carolina; UNC Chapel Hill, Chapel Hill, North Carolina; UNC Jaycee Burn Center, Chapel Hill, North Carolina; UNC Jaycee Burn Center, Chapel Hill, North Carolina; University of North Carolina at Chapel Hill, Chapel Hill, North Carolina; University of North Carolina Chapel Hill, Chapel Hill, North Carolina; UNC Chapel Hill, Dept of Surgery, Chapel Hill, North Carolina; UNC Jaycee Burn Center, Chapel Hill, North Carolina; Duke University Hospital, Durham, North Carolina; UNC Chapel Hill, Chapel Hill, North Carolina; UNC Jaycee Burn Center, Chapel Hill, North Carolina; UNC Jaycee Burn Center, Chapel Hill, North Carolina; University of North Carolina at Chapel Hill, Chapel Hill, North Carolina; University of North Carolina Chapel Hill, Chapel Hill, North Carolina; UNC Chapel Hill, Dept of Surgery, Chapel Hill, North Carolina; UNC Jaycee Burn Center, Chapel Hill, North Carolina; Duke University Hospital, Durham, North Carolina; UNC Chapel Hill, Chapel Hill, North Carolina; UNC Jaycee Burn Center, Chapel Hill, North Carolina; UNC Jaycee Burn Center, Chapel Hill, North Carolina; University of North Carolina at Chapel Hill, Chapel Hill, North Carolina; University of North Carolina Chapel Hill, Chapel Hill, North Carolina; UNC Chapel Hill, Dept of Surgery, Chapel Hill, North Carolina; UNC Jaycee Burn Center, Chapel Hill, North Carolina; Duke University Hospital, Durham, North Carolina; UNC Chapel Hill, Chapel Hill, North Carolina; UNC Jaycee Burn Center, Chapel Hill, North Carolina; UNC Jaycee Burn Center, Chapel Hill, North Carolina; University of North Carolina at Chapel Hill, Chapel Hill, North Carolina; University of North Carolina Chapel Hill, Chapel Hill, North Carolina; UNC Chapel Hill, Dept of Surgery, Chapel Hill, North Carolina

## Abstract

**Introduction:**

Patients with complicated wound care, rashes, desquamating skin disorders and necrotizing soft tissue infections are often transferred to burn centers due to the multi-disciplinary expertise in wound care despite the pathophysiology of burn injury being different. Our objective was to critically evaluate outcomes of patients admitted to our institution with skin disorders to patients with burn injury.

**Methods:**

Patients were identified using Institutional Burn Center registry, and linked to the clinical and administrative data. All adult patients admitted between January 1, 2012 and June 30, 2022 were eligible for inclusion. Demographics, length of stay (LOS), and mortality were evaluated. Bivariate logistic regression and inverse probability of treatment weighting (IPTW) was performed, and significance was accepted as p< 0.05.

**Results:**

We included 9,821 patients in the study, of whom 805 (8.2%) were admitted with skin disorders. These patients were more likely to be older, Black, of female sex, have longer ICU stays, be on mechanical ventilation, and have complications, p< 0.001. Unadjusted estimates show that patients with skin disorders had 3.84 higher odds of mortality compared to patients admitted for burn injury. However, after adjusting for age, involved body surface area, sex, race, intensive care unit days, mechanical ventilation, inhalation injury, and complications using IPTW, patients with skin disorders twice higher odds of mortality.

**Conclusions:**

Patients with skin disorders have increased morbidity and mortality compared to burn injured patients even when controlling for age, involved body surface area, sex, race, intensive care unit days, mechanical ventilation, inhalation injury, and complications when admitted to our burn unit. Further study is warranted to investigate and mitigate these outcome disparities in this patient population.

**Applicability of Research to Practice:**

This study may demonstrate that all skin conditions are not the same, and may warrant different specialties to improve and optimize outcomes.

